# Time from Cardiopulmonary Resuscitation Initiation to Prehospital Return of Spontaneous Circulation and Downstream Outcomes Among Adult Out-of-Hospital Cardiac Arrest Patients Achieving Field ROSC: A 2015–2023 Smart Advanced Life Support Registry Study

**DOI:** 10.3390/diagnostics16152301

**Published:** 2026-07-23

**Authors:** Dahyun Park, Sohyeon Chun, Gi Woon Kim, Min-Seong Kang, Han Bit Kim

**Affiliations:** 1 Department of Emergency Medicine, Soonchunhyang University Hospital Bucheon, Bucheon-si 14584, Republic of Korea; 138288@schmc.ac.kr (D.P.); 129815@schmc.ac.kr (S.C.); gwkim@schmc.ac.kr (G.W.K.); 2 Department of Emergency Medical Technology, Dongnam Health University, Suwon 16328, Republic of Korea; goodemt10@dongnam.ac.kr

**Keywords:** OHCA, ROSC, time-to-ROSC, neurological outcome, rearrest, prehospital resuscitation

## Abstract

**Background**: Prehospital return of spontaneous circulation (ROSC) is a widely used intermediate endpoint in out-of-hospital cardiac arrest (OHCA), but its downstream clinical meaning may differ according to when ROSC is achieved. **Methods**: We conducted a retrospective registry-based cohort study of adult non-traumatic OHCA patients treated with Smart Advanced Life Support (SALS) from 2015 to 2023 who achieved prehospital ROSC. The primary exposure was the low-flow interval, defined as the interval from CPR initiation to first prehospital ROSC, categorized a priori as <10, 10–14, 15–19, 20–24, 25–29, and ≥30 min. Multivariable logistic regression models estimated associations with good neurological recovery, prehospital rearrest, survival to hospital admission, and survival to discharge, adjusting for age, sex, witnessed arrest, bystander CPR, initial shockable rhythm, transport time interval, and region. **Results**: Among 19,156 adult SALS-treated OHCA patients, 4195 achieved prehospital ROSC; 3760 had valid low-flow interval values from 0 to 60 min and were included in the primary analysis. Good neurological recovery decreased from 582/1004 (58.0%) in the <10 min group to 17/249 (6.8%) in the ≥30 min group, while prehospital rearrest increased from 194/488 (39.8%) to 136/174 (78.2%). Compared with <10 min, the adjusted odds ratios for good neurological recovery were 0.368, 0.164, 0.135, 0.099, and 0.081 across progressively later ROSC categories. The adjusted odds ratio for rearrest in the ≥30 min group was 5.024. **Conclusions**: In this selected SALS-treated ROSC-positive OHCA cohort, later low-flow interval was associated with substantially lower odds of favorable neurological recovery and survival and higher odds of prehospital rearrest. These findings suggest that prehospital ROSC timing may serve as a prognostic marker but should not be interpreted as a standalone criterion for treatment termination or transport decisions.

## 1. Introduction

Prehospital return of spontaneous circulation (ROSC) is one of the most frequently reported intermediate endpoints in out-of-hospital cardiac arrest (OHCA) research and quality assessment, and Utstein-style reporting frameworks continue to treat ROSC and downstream survival outcomes as core descriptors of resuscitation performance [1]. In both clinical care and registry analyses, achievement of ROSC is often regarded as a sign of successful field resuscitation. However, ROSC is not a uniform endpoint. Some patients achieve early and durable ROSC with favorable downstream recovery, whereas others achieve ROSC only after prolonged resuscitation and remain vulnerable to rearrest, in-hospital death, or poor neurological outcome.

Prior studies of resuscitation duration have shown that favorable neurological outcome declines rapidly as CPR duration lengthens, and that most patients with good outcomes achieve ROSC relatively early in the resuscitation course [2,3,4]. Similarly, among patients who achieve prehospital ROSC, a shorter time-to-ROSC has been associated with higher short-term survival [5].

Although prolonged resuscitation is generally associated with worse outcomes, fewer studies have specifically examined the downstream clinical meaning of time to first prehospital ROSC among patients who actually achieve ROSC in the field. de Graaf et al. showed that shorter time-to-ROSC among patients with prehospital ROSC was strongly associated with 30-day survival and suggested that transport decisions without ROSC may need to occur relatively early in the resuscitation course [5]. Woo et al., in an SALS-treated OHCA cohort with prehospital ROSC, showed that rearrest after ROSC was common and that longer collapse-to-first-ROSC intervals were associated with rearrest [6]. Hospital-based post-ROSC studies have also shown that prolonged downtime does not make favorable neurological recovery uniformly impossible, even beyond conventional time thresholds, particularly in selected patients receiving targeted temperature management or other intensive post-resuscitation care [7,8].

However, these studies address adjacent rather than identical questions. The de Graaf study focused mainly on survival and transport timing, the Woo study on rearrest after prehospital ROSC, and the hospital-based prolonged-downtime studies on post-ROSC prognosis rather than the immediate prehospital meaning of first ROSC in an SALS setting. Existing evidence therefore remains fragmented, with neurological recovery, downstream survival, and prehospital rearrest having been evaluated separately rather than together according to ROSC timing within an SALS-treated cohort restricted to patients with prehospital ROSC [5,6,7,8].

Accordingly, we examined the association between time to first prehospital ROSC and multiple downstream outcomes in a large SALS-treated adult OHCA registry. We hypothesized that later ROSC would be associated with progressively lower good neurological recovery and lower survival, while being associated with greater rearrest vulnerability. Our objective was to clarify the prognostic significance of ROSC timing by evaluating these clinically relevant outcomes within a single analytic framework.

## 2. Methods

### 2.1. Study Design and Population

This retrospective registry-based cohort study included adult patients (≥18 years) with non-traumatic OHCA who were treated with smart advanced life support (SALS) between 2015 and 2023. This study was reported in accordance with the STROBE statement for observational studies. The source population for SALS has been described previously in detail [9]. In brief, SALS was implemented within designated Korean EMS regions for medical-cause OHCA patients considered eligible for physician-guided prehospital advanced life support. Patients could be excluded from SALS eligibility or receipt because of age < 18 years, obvious signs of death, pre-existing do-not-resuscitate status or refusal of CPR at the scene, incomplete records, arrest outside SALS target regions, immediate transport or physician-directed non-SALS management, or operational/clinical reasons that prevented SALS delivery. Even among SALS-eligible patients, SALS was not always performed in cases such as arrest during transport, failed physician video connection, guardian refusal, or physician judgment against SALS. Final SALS delivery depended on physician availability, operational feasibility, successful physician–EMS communication, and on-scene clinical judgment. In the present dataset, we could reliably identify the SALS-treated cohort and downstream analytic subsets, but we could not reconstruct the full OHCA source population or all excluded non-SALS cases at the same granularity. Because the registry used for the present study begins with SALS-treated patients, the numbers and characteristics of SALS-eligible patients who did not receive SALS, as well as the overall upstream OHCA population, were not available. Accordingly, the analytic flow for this study is presented from the SALS-treated cohort onward. For the present analysis, we restricted the cohort to patients who achieved prehospital ROSC because the study question focused on the downstream clinical meaning of ROSC timing after field ROSC had occurred. Patients without prehospital ROSC were not part of the primary analytic cohort. We further required a valid low-flow interval, defined as CPR initiation to first prehospital ROSC, and excluded values > 60 min from the primary analysis as extreme or potentially unreliable registry values. These excluded observations were evaluated in a sensitivity analysis.

### 2.2. SALS System Context

SALS is a Korean prehospital resuscitation model developed to support advanced life support delivery for OHCA in the field, particularly in regional EMS systems where prolonged on-scene resuscitation and structured physician oversight are feasible. In contrast to conventional rapid transport-oriented basic life support practice, SALS incorporates sustained on-scene advanced resuscitation, real-time physician guidance, and protocolized use of ALS-level interventions.

Operationally, SALS involves real-time medical oversight by emergency physicians, typically through video-assisted or app-based communication with emergency medical technicians (EMTs) in the field. Under physician guidance, EMS personnel may perform prolonged high-quality on-scene CPR, rhythm-based defibrillation, advanced airway management, and intravenous drug administration including epinephrine and amiodarone, while sharing clinical information such as electrocardiographic rhythm and end-tidal CO_2_ when available. The SALS framework was introduced as a pilot project in Korea in 2015 and expanded across multiple regions in collaboration with tertiary hospitals and fire stations. Detailed descriptions of the SALS operational model and its nationwide implementation have been reported previously [9,10].

An additional strength of the SALS dataset is the granularity and cross-verification of procedural documentation. The SALS record form captures major resuscitation procedures and time-stamped events in minute-level detail, which increases the reliability of elapsed-time variables and on-scene intervention histories. For analytic use, records were not based on a single source alone; instead, EMS run sheets, detailed cardiac arrest forms, and SALS procedure logs were merged and cross-checked. This multi-source reconciliation process reduced reliance on any single document and improved the credibility of key timing variables, including the recorded time of first prehospital ROSC, and intervention variables used in the present study. The cohort derivation was adult SALS-treated OHCA during 2015–2023, *n* = 19,156; prehospital ROSC achieved, *n* = 4195; valid low-flow interval from 0 to 300 min, *n* = 3771; excluded low-flow interval > 60 min, *n* = 11; final primary analytic cohort, *n* = 3760.

### 2.3. Exposure and Outcomes

The primary exposure was the low-flow interval, defined as the interval from CPR initiation to first prehospital ROSC, measured in minutes. In the registry codebook, this variable corresponds to Low-Flow Time, defined as CPR start to first ROSC. Therefore, this exposure should not be interpreted as collapse-to-ROSC, EMS call-to-ROSC, or scene-arrival-to-ROSC time. The exposure was categorized a priori as <10, 10–14, 15–19, 20–24, 25–29, and ≥30 min, using <10 min as the reference category. Values > 60 min were excluded from the primary analysis and examined in sensitivity analysis. These intervals were selected to preserve clinical interpretability and to reflect progressively delayed ROSC in a way that remains intuitive for prehospital resuscitation decision-making and reporting [2,3,4,5].

The primary outcome was good neurological recovery, defined as cerebral performance category (CPC) 1–2 at discharge, consistent with commonly reported OHCA outcome frameworks [1,3,4,11]. Secondary outcomes were prehospital rearrest, survival to hospital admission, and survival to hospital discharge. Prehospital rearrest was defined as recurrent loss of circulation after initial prehospital ROSC during the prehospital phase [6,12,13,14]. Rearrest analyses were conducted as available-case analyses because rearrest documentation was incomplete.

### 2.4. Statistical Analysis

Primary inferential analyses used multivariable logistic regression models with binomial likelihood and logit link for each outcome: good neurological recovery, prehospital rearrest, survival to hospital admission, and survival to hospital discharge. The primary predictor was low-flow interval category, with <10 min as the reference. Models were adjusted for age, sex, witnessed arrest, bystander CPR, initial shockable rhythm, transport time interval, and region. These covariates were selected as prognostic adjustment variables based on Utstein-style OHCA reporting and clinical relevance, rather than to estimate a causal effect of delaying ROSC [1,2,3,4,5,11,15,16,17,18].

Because the main clinical question concerned how delayed ROSC categories compared with early ROSC, categorical models were prespecified as the primary inferential approach. Restricted cubic spline models were used as exploratory visual analyses to depict continuous associations between low-flow interval and adjusted outcome probabilities. Spline figures included 95% confidence intervals and histograms of the observed low-flow interval distribution [2,3,4,5,15].

Effect-modifier analyses tested interactions between low-flow interval category and initial shockable rhythm, witnessed arrest, bystander CPR, and long transport time interval. Because some strata contained limited event counts, interaction findings were interpreted cautiously and supplemented with subgroup event-count tables. To preserve clinical interpretability and reduce optimism from future-state information, the primary inferential models did not use proxy-augmented or downstream-augmented machine-learning features. The multivariable logistic regression models were used to estimate adjusted associations rather than to develop a clinical prediction model. Therefore, formal assessment of model calibration was not performed. Sensitivity and diagnostic analyses included unadjusted models, models including patients with low-flow interval >60 min, models excluding transport time interval, models additionally adjusted for calendar year, rearrest missingness analyses, comparison of patients with versus without rearrest data (Supplementary Table S8), multicollinearity diagnostics using variance inflation factors, and a time-definition sensitivity analysis using raw first-ROSC time supplemented by CPR time (Supplementary Table S1). Pearson and Spearman correlations between low-flow interval and scene time interval were also calculated.

All analyses were performed in Python 3.14.3 (Python Software Foundation, Wilmington, DE, USA). The main packages were pandas 3.0.2, numpy 2.4.4, scipy 1.17.1, statsmodels 0.14.6, patsy 1.0.2, scikit-learn 1.8.0, matplotlib 3.10.8, and catboost 1.2.10.

## 3. Results

**Figure 1:**
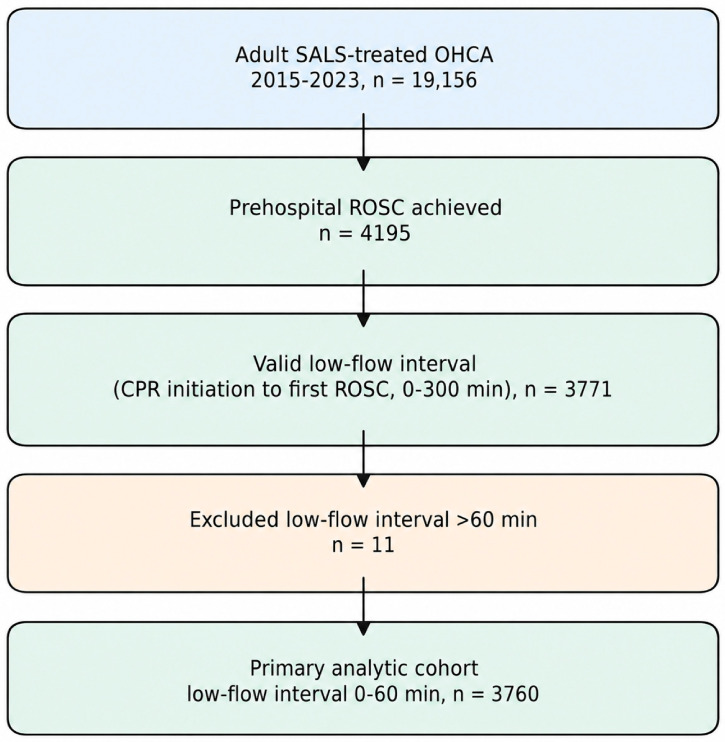
Study flow chart. Flow diagram showing cohort selection from adult SALS-treated OHCA patients in 2015–2023 to the primary analytic cohort. The primary exposure was the low-flow interval, defined as CPR initiation to first prehospital ROSC. Patients with low-flow interval >60 min were excluded from the primary analysis and evaluated in sensitivity analysis. SALS, Smart Advanced Life Support; OHCA, out-of-hospital cardiac arrest; ROSC, return of spontaneous circulation.

Figure 1 summarizes a derivation of the analytic cohort from the 2015–2023 adult SALS-treated OHCA registry. Among 19,156 adult SALS-treated OHCA patients, 4195 achieved prehospital ROSC. Of these, 3771 had a valid low-flow interval from CPR initiation to first ROSC between 0 and 300 min. After excluding 11 patients with low-flow interval >60 min, 3760 patients were included in the primary analytic cohort.

### 3.1. Baseline Characteristics of the Prehospital ROSC Cohort

Table 1 summarizes baseline characteristics according to low-flow interval category. Compared with patients achieving ROSC within 10 min, patients in later ROSC categories tended to be older, were less likely to have witnessed arrest, bystander CPR, an initial shockable rhythm, or arrest in a public location, and had longer scene time intervals. Pre-existing medical histories were also described, including hypertension, diabetes, prior stroke, heart disease, tuberculosis, liver cirrhosis proxy based on the hepatitis-coded field, and cancer. Outcome variables were removed from Table 1 and are reported separately in Table 2.

### 3.2. Downstream Outcomes According to Time to First Prehospital ROSC

A graded pattern was observed across low-flow interval categories. Good neurological recovery decreased from 582/1004 (58.0%) in the <10 min group to 17/249 (6.8%) in the ≥30 min group. Survival to discharge decreased from 713/1003 (71.1%) to 44/247 (17.8%), and survival to hospital admission decreased from 859/1002 (85.7%) to 140/246 (56.9%). In contrast, prehospital rearrest increased from 194/488 (39.8%) in the <10 min group to 136/174 (78.2%) in the ≥30 min group [2,3,4,5,15,16].

A total of 11 patients with low-flow interval > 60 min were excluded from the primary analysis. Their baseline profile and the sensitivity model including these observations are presented in the Supplementary Tables S2 and S4. The inclusion of these patients did not materially change the direction or interpretation of the associations.

### 3.3. Adjusted Association Between Later ROSC and Clinically Relevant Outcomes

Table 3 presents adjusted associations between low-flow interval category and the primary and secondary outcomes. Unadjusted results showed similar patterns (Supplementary Table S3). Compared with ROSC within 10 min, later low-flow interval categories were independently associated with progressively lower odds of good neurological recovery. Adjusted odds ratios for good neurological recovery were 0.368 (95% CI, 0.289–0.468) for 10–14 min, 0.164 (95% CI, 0.121–0.222) for 15–19 min, 0.135 (95% CI, 0.095–0.193) for 20–24 min, 0.099 (95% CI, 0.061–0.161) for 25–29 min, and 0.081 (95% CI, 0.046–0.143) for ≥30 min. Later ROSC was also associated with lower odds of survival to hospital admission and survival to discharge, and higher odds of prehospital rearrest. For prehospital rearrest, the adjusted odds ratio was 5.024 (95% CI, 3.237–7.797) in the ≥30 min group.

### 3.4. Figure-Based Depiction of the Continuous ROSC–Time Relationship

**Figure 2:**
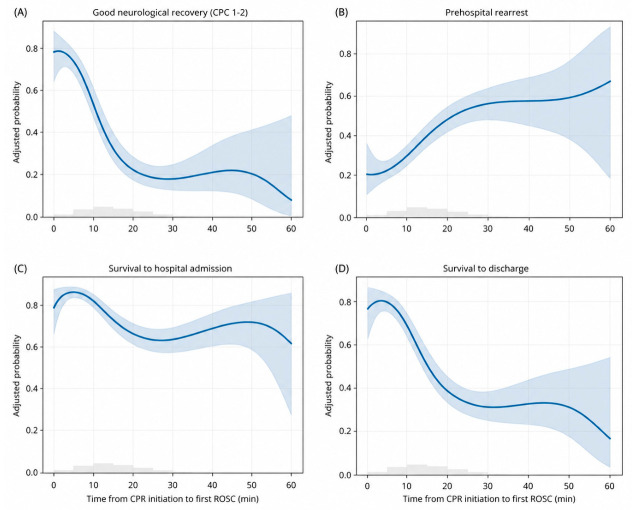
Exploratory restricted cubic spline curves for low-flow interval and downstream outcomes. Adjusted predicted probabilities are shown for (**A**) good neurological recovery, (**B**) prehospital rearrest, (**C**) survival to hospital admission, and (**D**) survival to hospital discharge according to low-flow interval, defined as CPR initiation to first prehospital ROSC. Shaded bands indicate 95% confidence intervals. Histograms show the observed distribution of low-flow interval. Models were adjusted for age, sex, witnessed arrest, bystander CPR, initial shockable rhythm, transport time interval, and region. CPC, cerebral performance category; CPR, cardiopulmonary resuscitation; ROSC, return of spontaneous circulation.

Figure 2 complements the prespecified categorical analyses by showing exploratory restricted cubic spline curves for the continuous association between low-flow interval and adjusted outcome probabilities. The curves demonstrated a steep decline in adjusted probabilities of good neurological recovery and survival as low-flow interval increased, whereas the adjusted probability of prehospital rearrest increased with longer low-flow interval. Confidence intervals widened at later time points where observations were fewer.

### 3.5. Effect-Modifier Analysis

Across prespecified effect modifiers, there was no consistent evidence that the association between low-flow interval and major outcomes differed substantially by initial shockable rhythm, witnessed arrest, bystander CPR, or transport burden. Because several interaction strata had limited event counts, these analyses were interpreted as exploratory. Event counts within interaction and subgroup strata are provided in Supplementary Table S10.

## 4. Discussion

In this registry-based analysis of SALS-treated adult OHCA patients with prehospital ROSC, the timing of first ROSC carried important prognostic meaning beyond the binary achievement of ROSC itself. Later ROSC was associated with a stepwise decline in the primary outcome of good neurological recovery, together with less favorable secondary outcomes, including lower survival to hospital admission, lower survival to discharge, and a substantially higher risk of prehospital rearrest. The principal implication of these findings is that prehospital ROSC should not be interpreted as a uniform marker of success. Rather, even among patients who achieve ROSC in the field, the time required to obtain ROSC provides additional prognostic information regarding downstream outcomes. These results should be interpreted within the selected responder cohort of patients who achieved prehospital ROSC during SALS. The findings do not estimate the probability of achieving ROSC among all OHCA patients and should not be generalized to patients who never achieved prehospital ROSC.

Achieving ROSC in the field remains clinically meaningful. However, our findings suggest that patients with later ROSC may represent a clinically distinct subgroup rather than simply patients who achieved ROSC later during resuscitation. The higher rearrest burden observed after delayed ROSC suggests greater post-resuscitation physiological instability, which may partly explain the poorer neurological and survival outcomes observed in these patients.

Most previous studies have focused on overall resuscitation duration or termination-of-resuscitation thresholds [2,3,4,10,15,19,20]. By contrast, the present study specifically examined patients who achieved prehospital ROSC and evaluated the prognostic significance of ROSC timing for downstream outcomes. In this respect, our findings suggest that prehospital ROSC should be interpreted in the context of its timing rather than simply as a binary endpoint in registry reporting and clinical communication [1,21].

These findings are consistent with previous studies showing that favorable neurological outcome decreases rapidly as resuscitation duration increases and that most patients with good outcomes achieve ROSC relatively early [2,3,4,5]. They also extend the observations of de Graaf et al., who reported that shorter time to prehospital ROSC was strongly associated with 30-day survival among patients achieving prehospital ROSC [5]. Our results are directionally concordant with that study, but add clinically relevant granularity by evaluating good neurological recovery and prehospital rearrest alongside downstream survival in an SALS-treated cohort.

Our data also align with the SALS-specific rearrest study by Woo et al., in which rearrest after prehospital ROSC was common and longer collapse-to-first-ROSC intervals were associated with rearrest [6]. In our analysis, the rearrest association remained strong and showed a clear gradient across later ROSC categories, supporting the interpretation that delayed ROSC may identify patients at higher risk of post-resuscitation instability. At the same time, our study moves beyond rearrest alone by showing how ROSC timing relates simultaneously to neurological recovery, survival to hospital admission, and survival to discharge.

A further comparison comes from post-ROSC hospital-based cohorts. Lee et al. and Ahn et al. showed that even after prolonged downtime, favorable neurological recovery is not uniformly absent in selected OHCA patients who reach the hospital and receive post-resuscitation care [7,8]. Those studies caution against overly rigid time-based nihilism, and our findings support the same general principle: late prehospital ROSC should not be interpreted as uniformly futile. However, our study addresses a different clinical question. Rather than asking whether prolonged total downtime after ROSC can still culminate in good outcomes under hospital-based care, we examined the prognostic significance of the timing of first prehospital ROSC in SALS-treated OHCA and its association with neurological recovery, survival, and prehospital rearrest.

From a clinical and systems perspective, these results support a more nuanced interpretation of ROSC as an outcome measure. Studies that treat all ROSCs equally may obscure clinically meaningful differences in patient trajectory. Time from CPR initiation to ROSC may therefore serve as an important contextual descriptor when comparing resuscitation performance, patient severity, and downstream outcomes. During prehospital-to-hospital handoff, ROSC timing may provide additional prognostic context and early post-resuscitation instability. However, ROSC timing should not be interpreted as a standalone criterion for termination of resuscitation, transport decisions, or withdrawal of post-resuscitation care [22]. Rather, it should be interpreted together with the overall clinical condition and other dynamic physiological markers, such as end-tidal carbon dioxide, which may provide complementary information during resuscitation, although further validation remains necessary [23].

Importantly, our findings should not be interpreted as indicating that late prehospital ROSC is futile. Although the prognostic value of ROSC decreases as time to ROSC increases, favorable neurological recovery remained possible in a subset of patients. The observed associations should not be interpreted as evidence that late ROSC is futile. Rather, later low-flow interval identifies a subgroup with substantially lower downstream probability of favorable recovery and higher post-ROSC instability. Clinical decisions regarding ongoing resuscitation, transport, or termination should incorporate the overall clinical context rather than ROSC timing alone.

Several limitations deserve emphasis. First, the study was retrospective and observational, and conditioning on prehospital ROSC creates a selected responder cohort [17,18]. This restriction may introduce selection bias or collider bias and prevents causal interpretation. Residual confounding is also possible despite multivariable adjustment. Second, the low-flow interval was operationalized from registry timing data and may be affected by documentation variability; it represents CPR initiation to first ROSC, not collapse-to-ROSC or EMS call-to-ROSC time. Third, rearrest analyses were available-case analyses because rearrest documentation was incomplete (Supplementary Table S7). If rearrest missingness was related to patient severity, EMS operational factors, or documentation practices, the estimated association with rearrest may be biased. Fourth, scene time interval was closely related to ROSC timing and was therefore not included in the primary model because scene time largely reflects ongoing resuscitation before ROSC and therefore overlaps conceptually with the exposure; diagnostic analyses showed Pearson and Spearman correlations of 0.662 and 0.705, respectively, between time to ROSC and scene time interval (Supplementary Table S11). Fifth, hospital-level factors and detailed in-hospital post-resuscitation interventions were not available in the prehospital registry. Sensitivity analyses including patients with low-flow interval >60 min, excluding transport time interval, and additionally adjusting for calendar year yielded materially similar findings (Supplementary Tables S4–S6). Variance inflation factor diagnostics did not indicate severe multicollinearity among covariates included in the primary adjusted models (Supplementary Table S9).

Despite these limitations, the study has notable strengths. It used a large contemporary SALS registry, focused on a clinically intuitive and operationally relevant exposure, and examined not only survival and neurological recovery but also post-ROSC instability through rearrest. This framework helps move the interpretation of ROSC beyond a simple yes/no endpoint toward a more clinically meaningful understanding of the post-ROSC trajectory. In practical terms, our findings support reporting and interpreting prehospital ROSC together with its timing and the broader clinical context rather than considering ROSC alone as a sufficient summary of early resuscitation success.

## 5. Conclusions

Among adult non-traumatic OHCA patients who achieved prehospital ROSC during SALS, later low-flow interval from CPR initiation to first prehospital ROSC was independently associated with substantially lower odds of good neurological recovery, survival to hospital admission, and survival to discharge, and higher odds of prehospital rearrest. ROSC timing may help characterize downstream prognosis among patients who achieve prehospital ROSC, but it should not be interpreted as a standalone criterion for termination of resuscitation, transport, or withdrawal of care decisions.

## Figures and Tables

**Table 1:** Baseline characteristics of the prehospital ROSC cohort according to low-flow interval category.

Characteristic	<10 min	10–14 min	15–19 min	20–24 min	25–29 min	≥30 min	Overall
Patients, *n*	1005	931	779	517	279	249	3760
Age, mean (SD), y	58.9 (15.0)	62.9 (16.0)	65.5 (15.6)	66.3 (15.4)	64.7 (16.8)	64.4 (15.5)	63.1 (15.8)
Female sex, *n*/N (%)	230/1005 (22.9%)	266/931 (28.6%)	256/779 (32.9%)	168/517 (32.5%)	91/279 (32.6%)	90/249 (36.1%)	1101/3760 (29.3%)
Witnessed arrest, *n*/N (%)	772/1003 (77.0%)	601/928 (64.8%)	475/774 (61.4%)	305/517 (59.0%)	167/279 (59.9%)	149/248 (60.1%)	2469/3749 (65.9%)
Bystander CPR, *n*/N (%)	794/1003 (79.2%)	658/927 (71.0%)	513/774 (66.3%)	318/516 (61.6%)	168/277 (60.6%)	145/244 (59.4%)	2596/3741 (69.4%)
Initial shockable rhythm, *n*/N (%)	701/1005 (69.8%)	345/931 (37.1%)	196/779 (25.2%)	149/517 (28.8%)	80/279 (28.7%)	61/249 (24.5%)	1532/3760 (40.7%)
Public location, *n*/N (%)	393/1005 (39.1%)	227/931 (24.4%)	205/779 (26.3%)	105/517 (20.3%)	50/279 (17.9%)	41/249 (16.5%)	1021/3760 (27.2%)
Hypertension, *n*/N (%)	275/1005 (27.4%)	281/931 (30.2%)	240/779 (30.8%)	173/517 (33.5%)	98/279 (35.1%)	59/249 (23.7%)	1126/3760 (29.9%)
Diabetes, *n*/N (%)	161/1005 (16.0%)	172/931 (18.5%)	169/779 (21.7%)	118/517 (22.8%)	68/279 (24.4%)	52/249 (20.9%)	740/3760 (19.7%)
Prior stroke, *n*/N (%)	46/1005 (4.6%)	65/931 (7.0%)	62/779 (8.0%)	35/517 (6.8%)	19/279 (6.8%)	17/249 (6.8%)	244/3760 (6.5%)
Heart disease, *n*/N (%)	215/1005 (21.4%)	171/931 (18.4%)	138/779 (17.7%)	99/517 (19.1%)	49/279 (17.6%)	39/249 (15.7%)	711/3760 (18.9%)
Tuberculosis, *n*/N (%)	1/1005 (0.1%)	1/931 (0.1%)	0/779 (0.0%)	0/517 (0.0%)	0/279 (0.0%)	2/249 (0.8%)	4/3760 (0.1%)
Liver cirrhosis proxy (hepatitis-coded), *n*/N (%)	1/1005 (0.1%)	2/931 (0.2%)	4/779 (0.5%)	0/517 (0.0%)	0/279 (0.0%)	0/249 (0.0%)	7/3760 (0.2%)
Cancer, *n*/N (%)	37/1005 (3.7%)	72/931 (7.7%)	69/779 (8.9%)	43/517 (8.3%)	17/279 (6.1%)	20/249 (8.0%)	258/3760 (6.9%)
Response time interval, median (IQR), min	7 (6–9)	7 (6–9)	7 (6–9)	7 (6–9)	8 (6–10)	8 (6–11)	7 (6–10)
Scene time interval, median (IQR), min	14 (11–17)	19 (16–21)	23 (20–25)	26 (22–29)	28 (22–33)	29 (25–36)	20 (16–25)
Transport time interval, median (IQR), min	7 (5–12)	7 (4–10)	7 (4–10)	7 (5–12)	8 (5–13)	10 (6–15)	7 (5–11)

Continuous variables are presented as mean (SD) or median (25th–75th percentile), and categorical variables as *n*/N (%). Data were missing for response time interval (*n* = 1), scene time interval (*n* = 3), and transport time interval (*n* = 4). Low-flow interval was defined as CPR initiation to first prehospital ROSC. Liver cirrhosis proxy was derived from the hepatitis-coded field because the original coding label was inconsistent with the intended clinical variable. CPR, cardiopulmonary resuscitation; IQR, interquartile range; ROSC, return of spontaneous circulation; SD, standard deviation.

**Table 2:** Primary and secondary outcomes according to low-flow interval category.

Outcome	Low-Flow Interval Category	Events/Non-Missing, *n*/N (%)
Good neurological recovery (CPC 1–2)	<10 min	582/1004 (58.0%)
	10–14 min	230/931 (24.7%)
	15–19 min	84/778 (10.8%)
	20–24 min	55/517 (10.6%)
	25–29 min	25/279 (9.0%)
	≥30 min	17/249 (6.8%)
Prehospital rearrest	<10 min	194/488 (39.8%)
	10–14 min	310/557 (55.7%)
	15–19 min	324/494 (65.6%)
	20–24 min	210/311 (67.5%)
	25–29 min	126/177 (71.2%)
	≥30 min	136/174 (78.2%)
Survival to hospital admission	<10 min	859/1002 (85.7%)
	10–14 min	675/931 (72.5%)
	15–19 min	456/777 (58.7%)
	20–24 min	280/514 (54.5%)
	25–29 min	136/276 (49.3%)
	≥30 min	140/246 (56.9%)
Survival to discharge	<10 min	713/1003 (71.1%)
	10–14 min	408/930 (43.9%)
	15–19 min	199/778 (25.6%)
	20–24 min	110/517 (21.3%)
	25–29 min	55/279 (19.7%)
	≥30 min	44/247 (17.8%)

Values are event count/non-missing denominator, *n*/N (%). Outcome-specific denominators differ because missingness was not identical across outcomes. Low-flow interval was defined as CPR initiation to first prehospital ROSC. CPC, cerebral performance category; ROSC, return of spontaneous circulation.

**Table 3:** Adjusted associations between low-flow interval category and outcomes.

Outcome	Low-Flow Interval Category	Adjusted OR (95% CI)	Overall *p* Value	Model *n*
Good neurological recovery (CPC 1–2)	<10 min	1.000 (reference)	<0.001	3725
	10–14 min	0.368 (0.289–0.468)		
	15–19 min	0.164 (0.121–0.222)		
	20–24 min	0.135 (0.095–0.193)		
	25–29 min	0.099 (0.061–0.161)		
	≥30 min	0.081 (0.046–0.143)		
Prehospital rearrest	<10 min	1.000 (reference)	<0.001	2172
	10–14 min	1.926 (1.469–2.525)		
	15–19 min	2.841 (2.130–3.790)		
	20–24 min	3.189 (2.298–4.425)		
	25–29 min	3.268 (2.191–4.874)		
	≥30 min	5.024 (3.237–7.797)		
Survival to hospital admission	<10 min	1.000 (reference)	<0.001	3713
	10–14 min	0.629 (0.493–0.803)		
	15–19 min	0.378 (0.296–0.483)		
	20–24 min	0.299 (0.229–0.390)		
	25–29 min	0.232 (0.169–0.319)		
	≥30 min	0.332 (0.238–0.463)		
Survival to discharge	<10 min	1.000 (reference)	<0.001	3721
	10–14 min	0.506 (0.409–0.625)		
	15–19 min	0.252 (0.199–0.319)		
	20–24 min	0.173 (0.131–0.228)		
	25–29 min	0.148 (0.104–0.212)		
	≥30 min	0.140 (0.095–0.206)		

Adjusted odds ratios were estimated using multivariable logistic regression, with low-flow interval <10 min as the reference category. Overall *p* values were obtained from likelihood-ratio tests comparing the full model containing the five low-flow interval indicator terms with the corresponding reduced model without those terms (5 degrees of freedom) and are reported once per outcome. Models were adjusted for age, sex, witnessed arrest, bystander CPR, initial shockable rhythm, transport time interval, and region. Model *n* differs by outcome because of outcome-specific missingness. CI, confidence interval; CPC, cerebral performance category; CPR, cardiopulmonary resuscitation; OR, odds ratio.

## Data Availability

The data presented in this study are not publicly available because of privacy and ethical restrictions.

## Supplementary Materials

The following supporting information can be downloaded at: https://www.mdpi.com/article/10.3390/diagnostics16152301/s1, Supplementary Table S1. Time-definition cohort comparison. Supplementary Table S2. Characteristics of patients included in the primary analysis and those excluded for low-flow interval >60 min. Supplementary Table S3. Unadjusted associations between low-flow interval category and outcomes. Supplementary Table S4. Sensitivity analysis including patients with low-flow interval >60 min. Supplementary Table S5. Sensitivity analysis excluding transport time interval from the adjustment set. Supplementary Table S6. Calendar-year adjusted sensitivity analysis. Supplementary Table S7. Rearrest data availability by low-flow interval category. Supplementary Table S8. Comparison of patients with and without rearrest data. Supplementary Table S9. Multicollinearity diagnostics for the adjusted models. Supplementary Table S10. Event counts in interaction and subgroup strata. Supplementary Table S11. Correlation between low-flow interval and scene time interval.
