# Supplementary material for: Time from Cardiopulmonary Resuscitation Initiation to Prehospital Return of Spontaneous Circulation and Downstream Outcomes Among Adult Out-of-Hospital Cardiac Arrest Patients Achieving Field ROSC: A 2015–2023 Smart Advanced Life Support Registry Study
Source: Diagnostics (Basel). 2026 Jul 23;16(15):2301. doi: 10.3390/diagnostics16152301 (PMC13465491; doi:10.3390/diagnostics16152301)
Supplement: Supplementary file 1 [file diagnostics-16-02301-s001.zip › Supplementary Tables.pdf]

Supplementary Tables

These tables are formatted for direct copy-and-paste into Microsoft Word. They use the corrected 2015-2023 SALS cohort and the primary exposure term low-flow interval (CPR initiation to first ROSC). Supplementary materials are numbered consistently as Supplementary Table 1 through Supplementary Table 11.

Supplementary Table S1. Time-definition cohort comparison

time definition	valid 0 300	excluded gt60	analytic le60
Primary: low-flow interval (CPR initiation to first ROSC)	3,771	11	3,760
Sensitivity: raw first ROSC time, supplemented by CPRtime	3,587	3	3,584

Note. Primary analysis used the low-flow interval, defined as the interval from CPR initiation to first ROSC. The raw/coalesced time definition was retained as a sensitivity cohort only.

Supplementary Table S2. Characteristics of patients included in the primary analysis and those excluded for low-flow interval >60 min

Group	n	Age mean	Female %	Shockable %	TTI median	Good CPC	Survival discharge	Rearrest
Included <=60 min	3,760	63.1	29.3	40.7	7.0	993/3758 (26.4%)	1529/3754 (40.7%)	1300/2201 (59.1%)
Excluded >60 min	11	62.9	36.4	45.5	13.0	2/11 (18.2%)	4/11 (36.4%)	9/10 (90.0%)

Note. Values are shown as n (%), mean (SD), or median (IQR), as applicable.

Supplementary Table S3. Unadjusted associations between low-flow interval category and outcomes

Outcome	Time to first ROSC	Unadjusted OR vs <10 min	95% CI lower	95% CI upper	P value	Model n
Good neurological recovery (CPC 1-2)	10-14	0.238	0.196	0.289	<0.001	3,758
Good neurological recovery (CPC 1-2)	15-19	0.088	0.068	0.114	<0.001	3,758
Good neurological recovery (CPC 1-2)	20-24	0.086	0.064	0.117	<0.001	3,758
Good neurological recovery (CPC 1-2)	25-29	0.071	0.046	0.110	<0.001	3,758
Good neurological recovery (CPC 1-2)	>=30	0.053	0.032	0.088	<0.001	3,758

Prehospital rearrest	10-14	1.902	1.486	2.434	<0.001	2,201
Prehospital rearrest	15-19	2.888	2.228	3.744	<0.001	2,201
Prehospital rearrest	20-24	3.151	2.337	4.248	<0.001	2,201
Prehospital rearrest	25-29	3.744	2.580	5.433	<0.001	2,201
Prehospital rearrest	>=30	5.424	3.626	8.114	<0.001	2,201
Survival to hospital admission	10-14	0.439	0.349	0.551	<0.001	3,746
Survival to hospital admission	15-19	0.236	0.188	0.297	<0.001	3,746
Survival to hospital admission	20-24	0.199	0.155	0.255	<0.001	3,746
Survival to hospital admission	25-29	0.162	0.120	0.217	<0.001	3,746
Survival to hospital admission	>=30	0.220	0.162	0.299	<0.001	3,746
Survival to discharge	10-14	0.318	0.263	0.384	<0.001	3,754
Survival to discharge	15-19	0.140	0.113	0.173	<0.001	3,754
Survival to discharge	20-24	0.110	0.086	0.141	<0.001	3,754
Survival to discharge	25-29	0.100	0.072	0.138	<0.001	3,754
Survival to discharge	>=30	0.088	0.062	0.126	<0.001	3,754

Note. Odds ratios are shown with 95% confidence intervals. The reference category is the earliest low-flow interval group unless otherwise specified in the table.

Supplementary Table S4. Sensitivity analysis including patients with low-flow interval >60 min

**Outcome**	**Time to first ROSC**	**Adjusted OR vs <10 min**	**95% CI lower**	**95% CI upper**	**P value**	**Model n**
Good neurological recovery (CPC 1-2)	10-14	0.368	0.289	0.469	<0.001	3,736
Good neurological recovery (CPC 1-2)	15-19	0.164	0.121	0.222	<0.001	3,736
Good neurological recovery (CPC 1-2)	20-24	0.135	0.095	0.193	<0.001	3,736
Good neurological recovery (CPC 1-2)	25-29	0.099	0.061	0.161	<0.001	3,736
Good neurological recovery (CPC 1-2)	>=30	0.085	0.050	0.147	<0.001	3,736
Prehospital rearrest	10-14	1.925	1.468	2.524	<0.001	2,182
Prehospital rearrest	15-19	2.843	2.131	3.792	<0.001	2,182
Prehospital rearrest	20-24	3.198	2.304	4.437	<0.001	2,182

Prehospital rearrest	25-29	3.283	2.201	4.897	<0.001	2,182
Prehospital rearrest	>=30	5.159	3.344	7.960	<0.001	2,182
Survival to hospital admission	10-14	0.630	0.494	0.805	<0.001	3,724
Survival to hospital admission	15-19	0.379	0.296	0.484	<0.001	3,724
Survival to hospital admission	20-24	0.299	0.229	0.391	<0.001	3,724
Survival to hospital admission	25-29	0.233	0.170	0.320	<0.001	3,724
Survival to hospital admission	>=30	0.343	0.247	0.477	<0.001	3,724
Survival to discharge	10-14	0.506	0.409	0.625	<0.001	3,732
Survival to discharge	15-19	0.252	0.199	0.319	<0.001	3,732
Survival to discharge	20-24	0.172	0.131	0.227	<0.001	3,732
Survival to discharge	25-29	0.148	0.104	0.212	<0.001	3,732
Survival to discharge	>=30	0.143	0.098	0.209	<0.001	3,732

Note. This analysis evaluates whether inclusion of patients with low-flow interval >60 min materially changed the estimated associations.

Supplementary Table S5. Sensitivity analysis excluding transport time interval from the adjustment set

**Outcome**	**Time to first ROSC**	**Adjusted OR vs <10 min**	**95% CI lower**	**95% CI upper**	**P value**	**Model n**
Good neurological recovery (CPC 1-2)	10-14	0.369	0.290	0.470	<0.001	3,729
Good neurological recovery (CPC 1-2)	15-19	0.164	0.121	0.223	<0.001	3,729
Good neurological recovery (CPC 1-2)	20-24	0.138	0.097	0.197	<0.001	3,729
Good neurological recovery (CPC 1-2)	25-29	0.100	0.062	0.163	<0.001	3,729
Good neurological recovery (CPC 1-2)	>=30	0.084	0.048	0.148	<0.001	3,729
Prehospital rearrest	10-14	1.891	1.444	2.477	<0.001	2,174
Prehospital rearrest	15-19	2.802	2.103	3.734	<0.001	2,174
Prehospital rearrest	20-24	3.206	2.312	4.445	<0.001	2,174
Prehospital rearrest	25-29	3.350	2.247	4.993	<0.001	2,174
Prehospital rearrest	>=30	5.180	3.343	8.027	<0.001	2,174
Survival to hospital	10-14	0.633	0.496	0.807	<0.001	3,717

admission						
Survival to hospital admission	15-19	0.382	0.299	0.488	<0.001	3,717
Survival to hospital admission	20-24	0.304	0.233	0.396	<0.001	3,717
Survival to hospital admission	25-29	0.237	0.173	0.325	<0.001	3,717
Survival to hospital admission	>=30	0.343	0.246	0.477	<0.001	3,717
Survival to discharge	10-14	0.507	0.410	0.627	<0.001	3,725
Survival to discharge	15-19	0.252	0.200	0.319	<0.001	3,725
Survival to discharge	20-24	0.176	0.133	0.231	<0.001	3,725
Survival to discharge	25-29	0.151	0.106	0.215	<0.001	3,725
Survival to discharge	>=30	0.145	0.098	0.213	<0.001	3,725

Note. This analysis was performed to assess whether adjustment for transport time interval influenced the association between low-flow interval and outcomes.

Supplementary Table S6. Calendar-year adjusted sensitivity analysis

**Outcome**	**Time to first ROSC**	**Adjusted OR vs <10 min**	**95% CI lower**	**95% CI upper**	**P value**	**Model n**
Good neurological recovery (CPC 1-2)	10-14	0.363	0.285	0.463	<0.001	3,725
Good neurological recovery (CPC 1-2)	15-19	0.161	0.119	0.219	<0.001	3,725
Good neurological recovery (CPC 1-2)	20-24	0.132	0.092	0.189	<0.001	3,725
Good neurological recovery (CPC 1-2)	25-29	0.096	0.059	0.158	<0.001	3,725
Good neurological recovery (CPC 1-2)	>=30	0.075	0.043	0.134	<0.001	3,725
Prehospital rearrest	10-14	1.953	1.449	2.633	<0.001	2,172
Prehospital rearrest	15-19	3.447	2.525	4.706	<0.001	2,172
Prehospital rearrest	20-24	3.859	2.706	5.504	<0.001	2,172
Prehospital rearrest	25-29	4.208	2.750	6.441	<0.001	2,172
Prehospital rearrest	>=30	7.018	4.413	11.163	<0.001	2,172
Survival to hospital admission	10-14	0.620	0.485	0.793	<0.001	3,713
Survival to hospital admission	15-19	0.371	0.290	0.476	<0.001	3,713

Survival to hospital admission	20-24	0.295	0.225	0.386	<0.001	3,713
Survival to hospital admission	25-29	0.235	0.171	0.324	<0.001	3,713
Survival to hospital admission	>=30	0.325	0.232	0.456	<0.001	3,713
Survival to discharge	10-14	0.498	0.402	0.616	<0.001	3,721
Survival to discharge	15-19	0.247	0.195	0.313	<0.001	3,721
Survival to discharge	20-24	0.168	0.127	0.222	<0.001	3,721
Survival to discharge	25-29	0.147	0.103	0.211	<0.001	3,721
Survival to discharge	>=30	0.137	0.092	0.202	<0.001	3,721

Note. Calendar year was additionally included to assess temporal changes across the study period.

Supplementary Table S7. Rearrest data availability by low-flow interval category

Time to first ROSC	Total n	Rearrest missing n (%)
<10	1,005	517/1005 (51.4%)
10-14	931	374/931 (40.2%)
15-19	779	285/779 (36.6%)
20-24	517	206/517 (39.8%)
25-29	279	102/279 (36.6%)
>=30	249	75/249 (30.1%)

Note. This table describes the availability of rearrest data across low-flow interval categories.

Supplementary Table S8. Comparison of patients with and without rearrest data

Group	n	Age mean	Female %	Shockable %	Good CPC %	Survival discharge %
Rearrest available	2,201	63.4	28.8	37.4	21.6	34.9
Rearrest missing	1,559	62.6	30.0	45.5	33.2	48.9

Note. This table evaluates potential differences between patients with available rearrest data and those with missing rearrest data.

Supplementary Table S9. Multicollinearity diagnostics for the adjusted models

Variable	VIF

t_rosc	1.855
STI	1.822
TTI	1.018
g_age	1.138
female	1.088
witnessed	1.061
bcpr	1.066
shockable	1.309

Note. Variance inflation factors were calculated for covariates included in the adjusted models. Values below commonly used thresholds suggested no severe multicollinearity.

Supplementary Table S10. Event counts in interaction and subgroup strata

**Subgroup**	**Level**	**Outcome**	**Events/non-missing (%)**	**Events**	**N**
Initial shockable rhythm	Yes/high	Good neurological recovery	862/1530 (56.3%)	862	1,530
Initial shockable rhythm	Yes/high	Survival to discharge	1049/1528 (68.7%)	1,049	1,528
Initial shockable rhythm	Yes/high	Prehospital rearrest	410/823 (49.8%)	410	823
Initial shockable rhythm	No/low	Good neurological recovery	131/2228 (5.9%)	131	2,228
Initial shockable rhythm	No/low	Survival to discharge	480/2226 (21.6%)	480	2,226
Initial shockable rhythm	No/low	Prehospital rearrest	890/1378 (64.6%)	890	1,378
Witnessed arrest	Yes/high	Good neurological recovery	777/2467 (31.5%)	777	2,467
Witnessed arrest	Yes/high	Survival to discharge	1133/2465 (46.0%)	1,133	2,465
Witnessed arrest	Yes/high	Prehospital rearrest	807/1419 (56.9%)	807	1,419
Witnessed arrest	No/low	Good neurological recovery	214/1280 (16.7%)	214	1,280
Witnessed arrest	No/low	Survival to discharge	392/1278 (30.7%)	392	1,278
Witnessed arrest	No/low	Prehospital rearrest	489/772 (63.3%)	489	772
Bystander CPR	Yes/high	Good neurological recovery	789/2595 (30.4%)	789	2,595
Bystander CPR	Yes/high	Survival to discharge	1156/2593 (44.6%)	1,156	2,593
Bystander CPR	Yes/high	Prehospital rearrest	843/1466 (57.5%)	843	1,466
Bystander CPR	No/low	Good neurological recovery	203/1144 (17.7%)	203	1,144
Bystander CPR	No/low	Survival to discharge	369/1142 (32.3%)	369	1,142
Bystander CPR	No/low	Prehospital rearrest	447/717 (62.3%)	447	717

Note. Event counts are provided to support interpretation of interaction and subgroup analyses.

Supplementary Table S11. Correlation between low-flow interval and scene time interval

**Diagnostic**	**Value**
Pearson correlation tROSC-STI	0.662
Spearman correlation tROSC-STI	0.705

Note. This diagnostic supports interpretation of potential correlation between low-flow interval and scene time interval.
